# Differences in proteome perturbations caused by the *Wolbachia* strain *w*Au suggest multiple mechanisms of *Wolbachia*-mediated antiviral activity

**DOI:** 10.1038/s41598-023-38127-4

**Published:** 2023-07-20

**Authors:** Stephanie M. Rainey, Vincent Geoghegan, Daniella A. Lefteri, Thomas H. Ant, Julien Martinez, Cameron J. McNamara, Wael Kamel, Zaydah Rolande de Laurent, Alfredo Castello, Steven P. Sinkins

**Affiliations:** 1 MRC-University of Glasgow-Centre for Virus Research, Glasgow, UK; 2 Present Address: The University of York, York, UK

**Keywords:** Viral vectors, Entomology, Protein-protein interaction networks

## Abstract

Some strains of the inherited bacterium *Wolbachia* have been shown to be effective at reducing the transmission of dengue virus (DENV) and other RNA viruses by *Aedes aegypti* in both laboratory and field settings and are being deployed for DENV control. The degree of virus inhibition varies between *Wolbachia* strains. Density and tissue tropism can contribute to these differences but there are also indications that this is not the only factor involved: for example, strains *w*Au and *w*AlbA are maintained at similar intracellular densities but only *w*Au produces strong DENV inhibition. We previously reported perturbations in lipid transport dynamics, including sequestration of cholesterol in lipid droplets, with strains *w*Mel/*w*MelPop in *Ae. aegypti.* To further investigate the cellular basis underlying these differences, proteomic analysis of midguts was carried out on *Ae. aegypti* lines carrying strains *w*Au and *w*AlbA: with the hypothesis that differences in perturbations may underline *Wolbachia*-mediated antiviral activity. Surprisingly, *w*Au-carrying midguts not only showed distinct proteome perturbations when compared to non-*Wolbachia* carrying and *w*AlbA-carrying midguts but also *w*Mel-carrying midguts. There are changes in RNA processing pathways and upregulation of a specific set of RNA-binding proteins in the *w*Au-carrying line, including genes with known antiviral activity. Lipid transport and metabolism proteome changes also differ between strains, and we show that strain *w*Au does not produce the same cholesterol sequestration phenotype as *w*Mel. Moreover, in contrast to *w*Mel, *w*Au antiviral activity was not rescued by cyclodextrin treatment. Together these results suggest that *w*Au could show unique features in its inhibition of arboviruses compared to previously characterized *Wolbachia* strains.

## Introduction

The maternally inherited intracellular symbiotic bacteria *Wolbachia* are common in insects and can spread through insect populations by inducing reproductive manipulations including cytoplasmic incompatibility (CI), a sperm modification that results in a pattern of crossing sterility that gives *Wolbachia-*carrying females a relative fitness advantage^1–3^. They are not naturally carried by the mosquito *Aedes aegypti*, the primary vector of the flaviviruses dengue (DENV), Zika (ZIKV) and yellow fever (YFV), and the alphavirus chikungunya (CHIKV), which together impose a huge public health burden across the tropics^4,5^. However, following lab transfers of various *Wolbachia* strains into this mosquito, some strains can reduce the transmission of DENV, ZIKV, YFV and CHIKV; *Wolbachia* can also inhibit insect-specific flaviviruses, West Nile Virus and Semliki Forest virus (SFV)^6–13^.

A number of studies have shown that the intracellular density of *Wolbachia* is an important factor in determining the relative ability of *Wolbachia* strains to inhibit viruses^13–16^. However, there have been recent indications that density is not the only factor involved: transfer of the *w*Au and *w*AlbA strains, originating in *Drosophila simulans* and *Aedes albopictus* respectively, into *Ae. aegypti* resulted in high intracellular densities in both cases, but *w*AlbA produced only limited antiviral activity against DENV/SFV and a relatively weak capacity to inhibit ZIKV in vivo^8,17^. In contrast *w*Au produced extremely efficient virus transmission blocking, with no evidence of any DENV dissemination beyond the midgut^8^*.* Mechanistically, a role has been demonstrated for lipid transport and metabolism in the ability of the *w*Mel/*w*MelPop strains (originating in *Drosophila melanogaster*) to inhibit DENV in vivo and in vitro in *Ae. aegypti*. An increase in cholesterol sequestration to lipid droplets occurs in *w*Mel/*w*MelPop-carrying *Ae. aegypti* cells, and treatment with the cyclodextrin 2HPCD released this stored cholesterol and induces a partial recovery of DENV replication^18^. However, it has not been examined whether these changes occur for all virus-inhibiting strains of *Wolbachia*.

Release programs using *Wolbachia*-carrying *Ae. aegypti* for DENV transmission control are underway in a number of countries^2,19,20^, using strain *w*Mel, or strain *w*AlbB originating in *Aedes albopictus.* An intervention trial using *w*AlbB in Malaysia showed 40–80% reduction in dengue incidence over multiple release sites^19^. With the continued field deployment of *Wolbachia* it is increasingly important to understand the molecular mechanisms underlying *Wolbachia*-mediated antiviral activity. Knowledge of the viral inhibition mechanisms will allow more informed monitoring and mitigation of potential operational problems, such as the possibility of viral ‘escape’ mutations or the instability of particular strains of the symbiont in given environments. When *Ae. aegypti* larvae are reared at temperatures above ~ 35 °C the density and maternal transmission of *w*Mel is lowered—potentially compromising its capacity to inhibit DENV in hot conditions and potentially elevating the risk of selection of escape mutations^8,21–25^. If *Wolbachia* strains can be identified for use in release programs that have mechanistic differences to *w*Mel/*w*AlbB in their viral inhibition, this would be highly valuable for long-term success of the strategy, in providing a means to either reduce the risk of selection of viral escape mutations, and/or allow a means of mitigation against viral escape should it occur.

In light of the unusually efficient viral inhibition conferred by strain *w*Au, which does not seem to be a consequence solely of its relatively high intracellular density^8,17^, we sought to examine whether any differences could be identified relative to other *Wolbachia* strains in terms of the cellular perturbations that may underlie virus inhibition. Proteomic analyses were utilized to compare the effects of *Wolbachia* strains in *Ae. aegypti* dissected midgut tissues^18^, and follow-up experiments carried out in cell culture.

## Results

### *w*Au induces distinct changes in protein expression

*Ae. aegypti* lines carrying *Wolbachia* strains *w*Au and *w*AlbA were chosen for comparative proteomic analyses as these two strains are found at similar intracellular densities in midguts following transfer into this host, but show contrasting levels of anti-viral activity, with *w*Au a much more efficient inhibitor of arbovirus transmission than *w*AlbA^8,17^ (Figure S1). Proteomic analysis was carried out on age-matched female midguts of *w*Au, *w*AlbA and wildtype *Wolbachia*-free (wt) *Ae. aegypti* in the same genetic background. Midguts were chosen as midgut cells are the site of initial arbovirus entry and replication in the mosquito, and previous proteomic analysis had shown results obtained from these tissues were robust for the study of *Wolbachia*/viral interactions^18^. In total, 3821 proteins were detected from all samples, of which 27 were identified as *Wolbachia* proteins, which were subsequently excluded from the KEGG pathway analysis as StringDB analysis is species specific. From the total proteins identified, 3379 were quantified in all sample groups and were therefore used for differential expression analysis.

**Figure 1:**
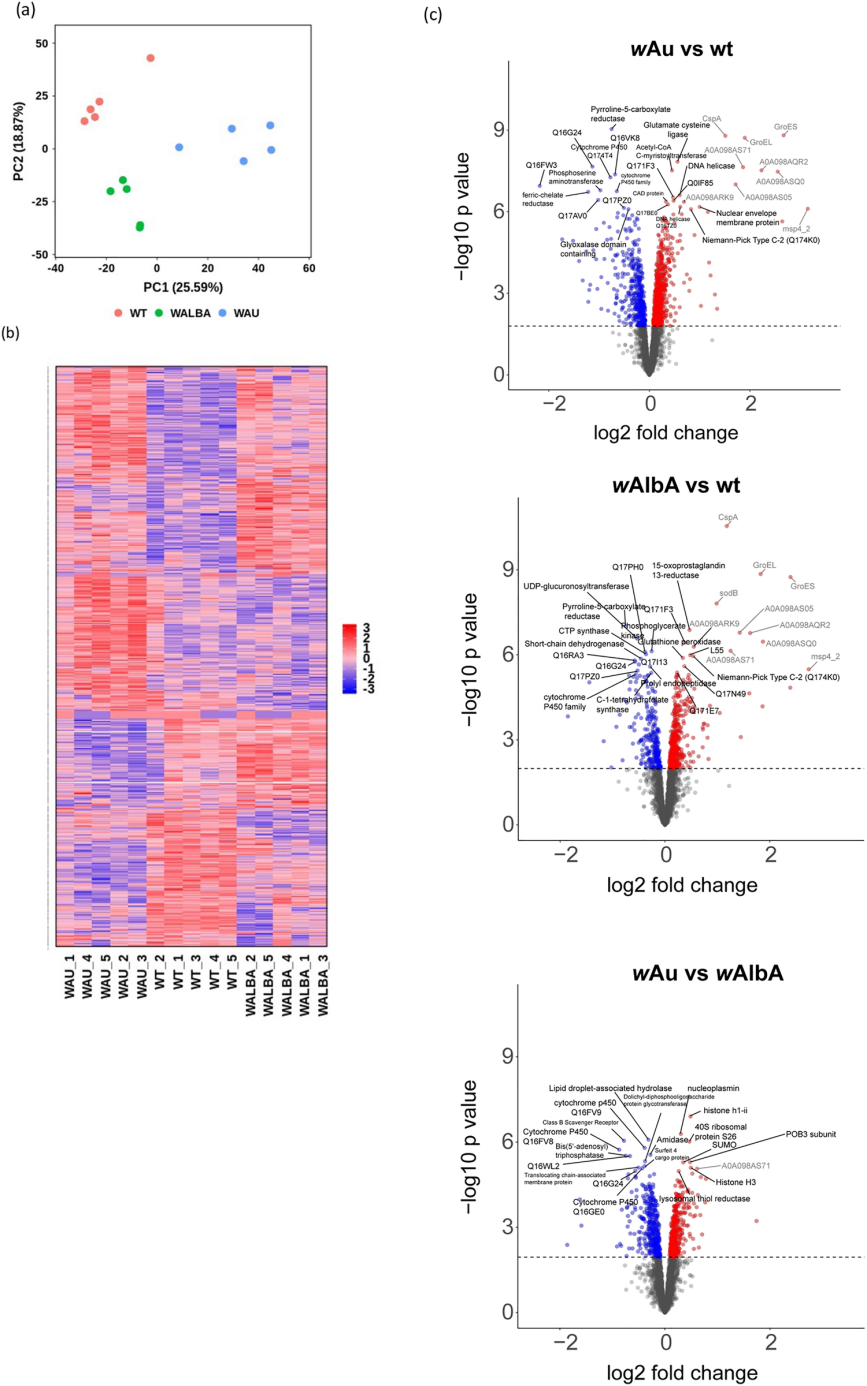
Comparison of proteins differentially expressed in *w*Au, *w*AlbA and wt midguts of *Aedes aegypti*. Pooled midguts were analysed by mass spectrometry to determine proteins that show significant alterations in expression between midguts containing *w*Au, *w*AlbA and wt (uninfected) N = 5. (**a**) Principal component analysis of all quantified proteins, each dot represents a single replicate. (**b**) Clustered heatmap of significantly dysregulated proteins when *w*Au or *w*AlbA are present compared to wt. (**c**) mass spectrometry quantitation of 3379 proteins identified from midguts of *Ae. aegypti*. Proteins present at significantly different levels are highlighted in red (up regulated) or blue (down regulated), dashed line denotes p-value threshold for a false discovery rate of 5%. Top 20 differentially regulated *Aedes* proteins are labelled, with most enriched *Wolbachia* proteins in grey.

A principal component analysis performed on protein expression levels generated a clear separation of biological replicates according to *Wolbachia* status/strain (Fig. 1a); differences in protein expression profiles could also be readily visualised in a heatmap representation of quantified proteins (Fig. 1b). A linear modelling-based approach to differential expression analysis detected the greatest level of dysregulation in *w*Au/wt with 1088 significantly different proteins, followed by *w*Au/*w*AlbA with 765 dysregulated proteins and *w*AlbA/wt with 706 dysregulated proteins at 5% FDR (Fig. 1c, Supplementary dataset). Volcano plot analysis of the differentially expressed proteins within each comparison shows a clear distinction between both *Wolbachia* strains and wt midguts and between the two strains. Relative abundance of the *Wolbachia* proteins detected above background indicate similar densities of *w*Au and *w*AlbA (Figure S2), confirming the suitability of this system to study density-independent differences in the level of antiviral activity between *Wolbachia* strains.

### Pathway analyses

**Figure 2:**
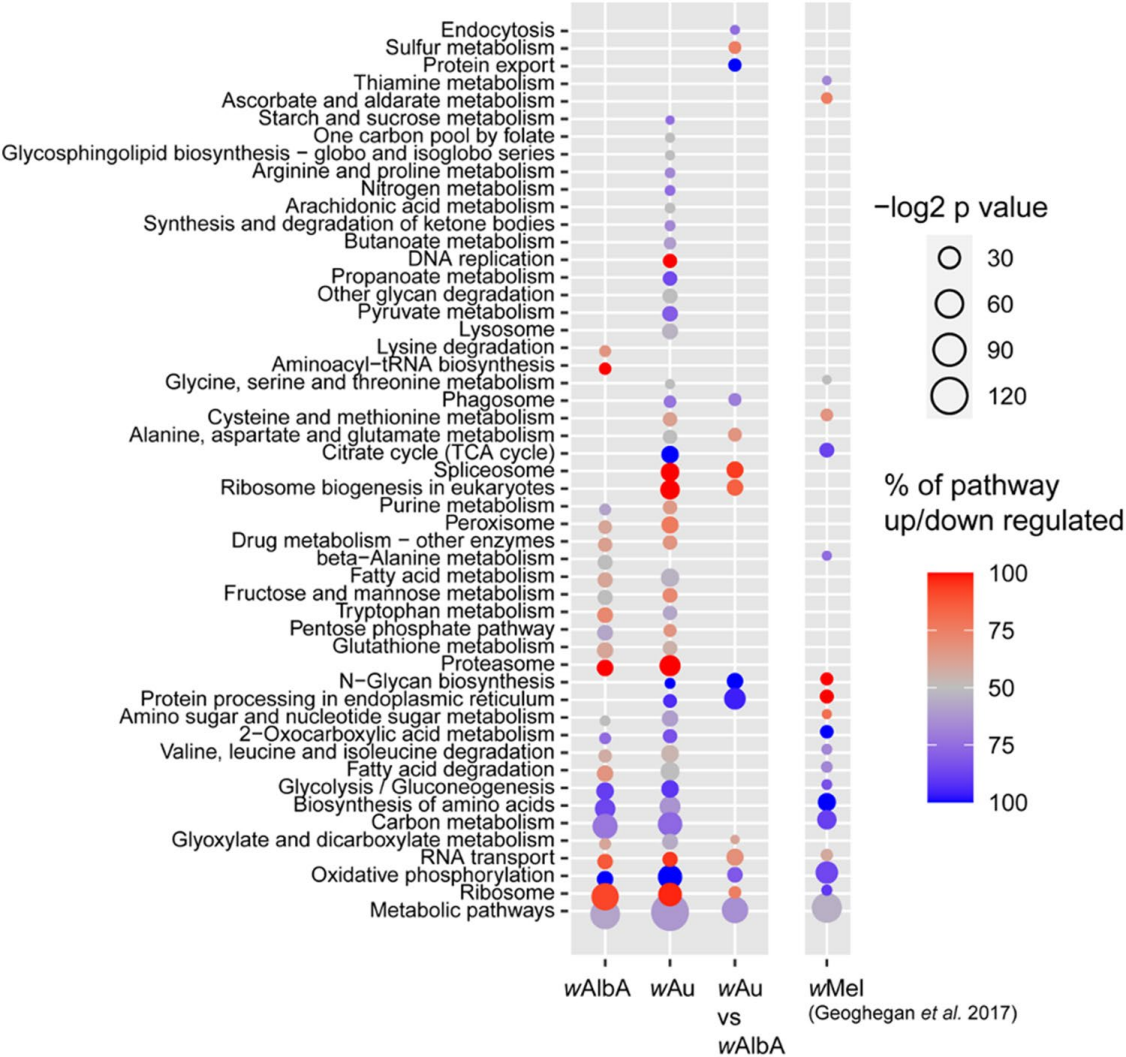
KEGG pathway analysis of differentially regulated proteins and pathway enrichment analysis based on *Wolbachia* strain-specific changes. Significantly dysregulated host proteins (FDR < 5%) were used to calculate over-represented KEGG pathway terms. Dysregulation is relative to uninfected midguts except the *w*Au vs *w*AlbA comparison. Bubble size is proportional to the statistical significance of the KEGG pathway enrichment. Proportion of each KEGG pathway up or down regulated is indicated with red or blue, respectively. The percentage is inclusive of all proteins in a given pathway, therefore taking into account those not detected in the dataset or not found to be significant.

Since there is a large dynamic response to *Wolbachia*, a global analysis was undertaken using the StringDB database^26^ in order to identify dysregulated pathways. The *Wolbachia*-carrying lines were compared to examine all proteins significantly dysregulated relative to the corresponding wt midguts. Proteins significantly dysregulated in *w*Au-carrying midguts relative to *w*AlbA midguts were also examined. A KEGG pathway analysis was conducted to examine the significantly over-represented pathways amongst the differentially regulated host proteins in each *Wolbachia* type, and our previously published dataset comparing *w*Mel and wt midguts was also analysed^18^ (Fig. 2). A direct comparison of *w*Mel to *w*Au/*w*AlbA was not undertaken due to the proteomic datasets having been generated at different times (although using the same protocols, instruments and personnel); instead, each dataset from lines carrying a different *Wolbachia* strain was compared to the wt *Wolbachia*-free control used in the same experiment, pathway analyses undertaken, and then comparisons made between the results.

Several pathways known to be involved in *Wolbachia* growth and metabolism were dysregulated in all *Wolbachia*-carrying lines, as expected. Genes involved in fatty acid synthesis and amino acid synthesis are absent from the genome of *Wolbachia*; therefore these processes are dysregulated when the bacterium is present^27–31^. The upregulation of proteasome proteins may be indicative of the need for a controlled breakdown of host proteins to increase amino acid availability^32^. However, there were marked differences between the three *Wolbachia*-carrying lines and in particular, between *w*Au and *w*Mel. The *w*Au containing midguts have a broader profile of pathway dysregulation compared to the *w*AlbA and *w*Mel midguts, for example affecting lysosomes, DNA replication and glycan degradation. To separate out host cellular pathway alterations that may be specifically associated with antiviral activity of *w*Au *Wolbachia* from the general ‘background’ perturbations caused by the presence of the bacterium, *Ae. aegypti* proteins significantly dysregulated in *w*Au midguts relative to *w*AlbA midguts were analysed for enrichment of KEGG pathways (Fig. 2). N-Glycan biosynthesis, protein processing in endoplasmic reticulum, ribosome biogenesis, protein export and endocytosis are significantly dysregulated in *w*Au compared to *w*AlbA midguts, all of which are known to be important for viral replication. Pathways involving RNA, RNA transport and the spliceosome were significantly affected which may directly affect the ability of viral RNA to replicate efficiently in the cell.

### Dynamics of *w*Au and *w*AlbA dysregulation of proteins and subdivision into criteria

To further characterise pathways that may be responsible for the greater antiviral activity associated with *w*Au compared to *w*AlbA, additional KEGG pathway enrichment analyses were carried out, this time by subdividing into 3 criteria: proteins that are either specifically dysregulated with *w*Au, specifically dysregulated with *w*AlbA or dysregulated in both strains but in opposite directions. More proteins were found to be upregulated compared to downregulated in the first two criteria. A considerably higher number of proteins were found to be significantly dysregulated specifically in the *w*Au line (409 proteins and 35 KEGG pathways) compared to *w*AlbA (230 proteins and 14 KEGG pathways), suggesting that *w*Au has a greater impact on the host proteome (Figure S3). Perturbed pathways already known to be involved in arbovirus replication are discussed below.

### RNA pathways and translation initiation are specifically enriched in *w*Au

Since DENV and other RNA viruses rely on host cell machinery to replicate, RNA pathway disruption may be important for *Wolbachia*-mediated antiviral activity^33,34^. Of the 35 KEGG pathways enriched only in *w*Au, 8 pathways are associated with RNA, DNA and splicing. Of the proteins dysregulated in these pathways all are upregulated in the *w*Au line compared to wt midguts. For comparison these proteins are not significantly dysregulated with *w*AlbA and show differing results in *w*Mel—for example, ribosomal proteins are downregulated with *w*Mel. These results indicate that there is a marked increase in RNA processing activity with *w*Au compared to the *Wolbachia*-negative line and lines carrying the *w*AlbA and *w*Mel strains. By comparing the pathways opposingly dysregulated in *w*Au and *w*AlbA midguts, it can be clearly seen that ribosome biogenesis is significantly enriched in *w*Au but significantly downregulated in *w*AlbA.

### RNA-binding proteins are distinctly dysregulated in *w*Au

**Figure 3:**
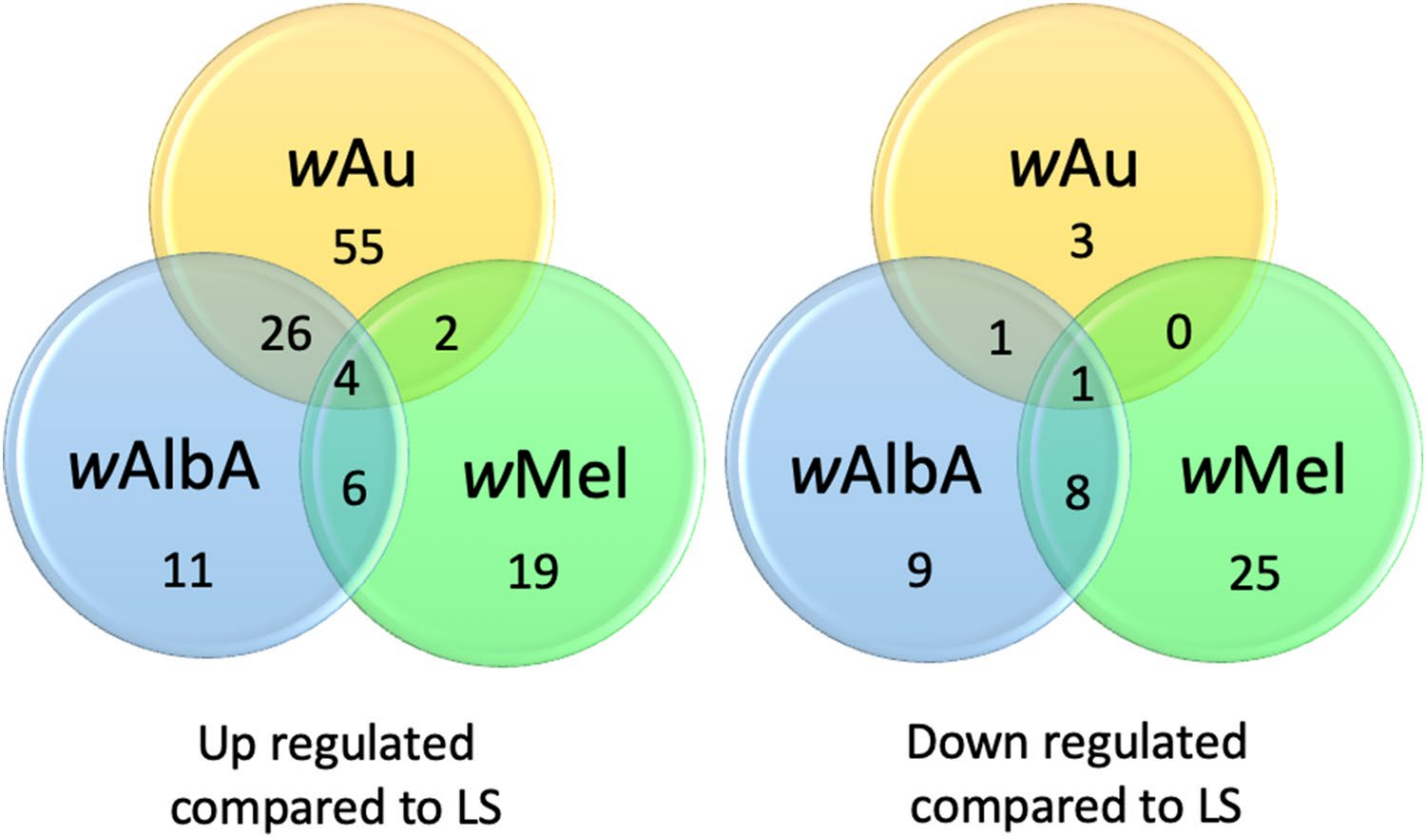
Proportion of perturbated proteins shown to have RNA binding capacity. Venn diagram showing the proportion of RNA binding proteins perturbed in the presence of each *Wolbachia* strain. Proteomic data was compared to an RNA Interactome (RIC) dataset to identify candidate RBPs. Proportion of each, direction of perturbations and overlapping patterns are presented.

RNA-binding proteins (RBPs) are known to be vital for the life cycle of positive sense RNA viruses^35–41^. To determine the RBPs dysregulated in midguts containing *Wolbachia*, proteomic data from this study was compared to pilot data resulting from an RNA interactome capture (RIC) study carried out in *Aedes* cells (A. Castello, W. Kamel, Z. Rolande de Laurent unpublished). Comparison of the pilot study to our proteomics datasets showed that of the 1088 significantly perturbed proteins between *w*Au/wt, 92 are RBPs; for *w*Mel/wt of the 434 perturbed proteins, 65 are RBPs; and for *w*AlbA/wt of the 706 perturbed proteins, 66 are RBPs (Fig. 3). Of the RBPs significantly perturbed in *w*Au midguts, 94% are upregulated compared to 71% for *w*AlbA and 47% for *w*Mel, again suggesting that *w*AlbA sits intermediate between *w*Au and *w*Mel. Although all strains show perturbations in RBPs there is very little overlap between *w*Mel and *w*Au. Further analysis of RBPs significantly dysregulated in *w*Au can be seen in Table S1. Several of the RBPs perturbed in *w*Au are not found in the *w*Mel dataset. However, of those found in both datasets, RBPs dysregulated in *w*Au are consistently either not significantly dysregulated in *w*Mel or are dysregulated in the opposite direction. Of the RBPs significantly dysregulated in *w*Au several are already known to bind DENV RNAs. Of the genes upregulated solely in *w*Au-carrying midguts, aBravo (AAEL004699) is known to show antiviral activity^41^, and the DEAD-box RNA helicases AAEL001216 and AAEL004859 were recently identified as among the top antiviral candidate RBPs from a RNAi screen targeting predicated RBPs in mosquito cells^35^. Intriguingly AAEL004859 exhibited an antiviral effect against multiple arboviruses. Altogether this supports the hypothesis that perturbations in RBPs may be important in *Wolbachia w*Au-mediated antiviral activity^36–42^.

### ER functions and trafficking

As highlighted in Table S1*w*Au and *w*AlbA lines show alterations in proteins associated with Endoplasmic Reticulum (ER) function and the unfolded protein response—important pathways in viral replication—that differ from *w*Mel. These pathways have previously been shown to be markedly perturbed in midguts containing *w*Mel^18^, but in the *w*Au line (Table S1), downregulation of some of the proteins that were upregulated in the *w*Mel line was observed, and no significant difference in others. Interestingly, the *w*AlbA line showed changes broadly intermediate between *w*Mel and *w*Au. The *w*Au line shows reduced glucuronosyltransferase and glycosyltransferase activity compared to *w*AlbA. Further affected processes involving trafficking and protein processing included the ECM receptor pathway, which was significantly upregulated in the *w*Au line but not with the other two strains. In mosquitoes this pathway is known to be involved in the stability of the extracellular matrix in the midgut and may play a role in the midgut infection barrier^42^. Of the pathways specific to *w*AlbA the only pathways that are not also enriched in *w*Mel are endocytosis and protein export, both of which are involved in arbovirus entry and replication/assembly.

### Lipid metabolism and transport

Of particular note, comparison of the *w*Au and *w*AlbA datasets showed marked differences in the perturbation of proteins involved in lipid metabolism and transport. Lipid dysregulation has previously been shown to be important in the inhibition of DENV in mosquito cells containing *w*Mel/*w*MelPop^18^. The proteome datasets generated here were therefore also compared to the previous dataset obtained from *w*Mel-carrying midguts^18^ (*w*Mel is found at lower intracellular density in *Ae. aegypti* midguts than either *w*Au or *w*AlbA, but is intermediate between the two in terms of virus inhibition). Perturbations in proteins associated with lipid homeostasis and lipid metabolism in the *w*Mel line were not seen in the *w*Au dataset (Table S1), where *w*Au is consistently different to *w*Mel.

### Cholesterol accumulation in lipid droplets and effects of cyclodextrin

**Figure 4:**
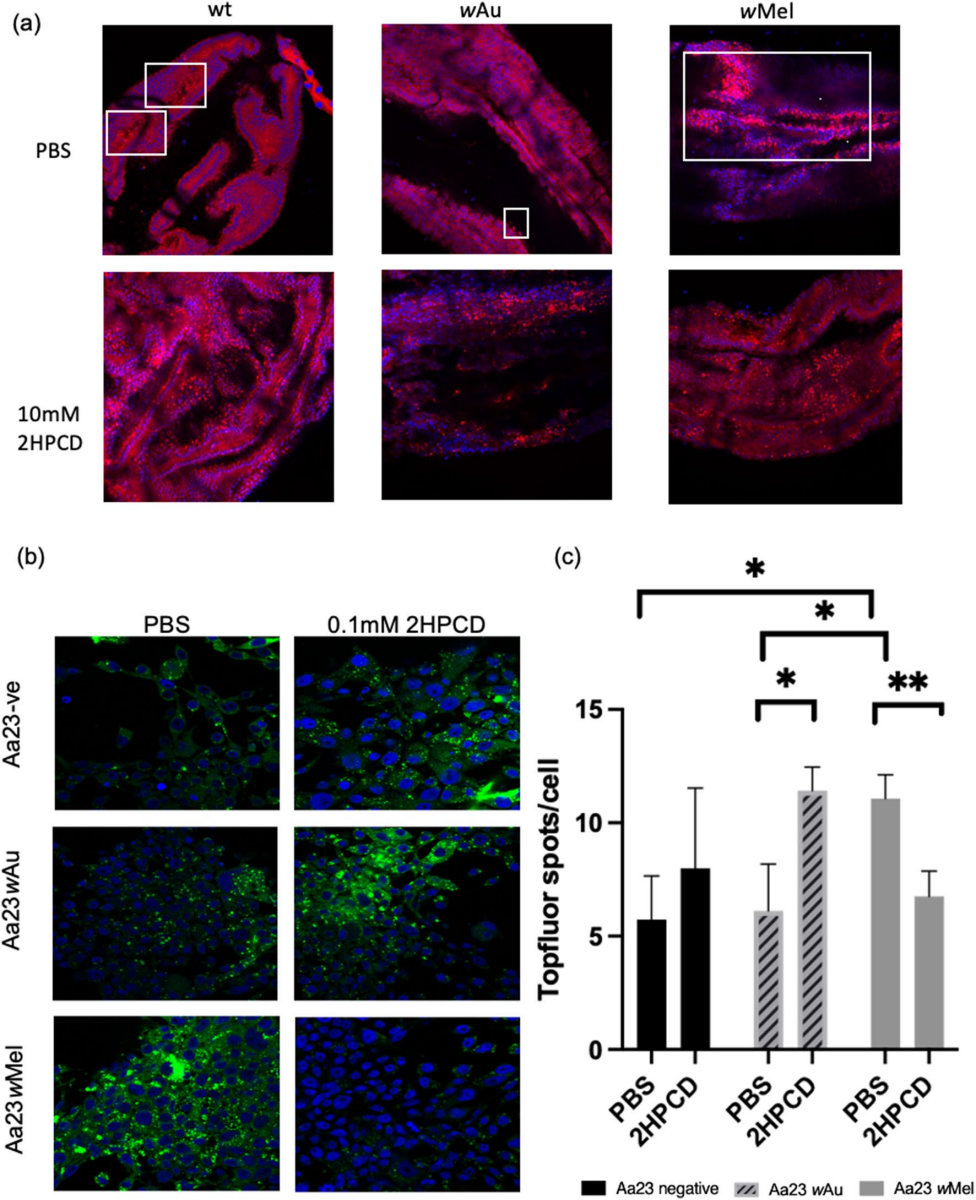
Effect of 2HPCD treatment on cholesterol dynamics in *Ae. aegypti* midguts and *Ae. albopictus* cell lines. (**a**) *Ae. aegypti* mosquitoes were injected with 10 mM of 2HPCD or PBS, left to recover for 2 days before a bloodmeal was given. 72 h post bloodmeal midguts were dissected, fixed and stained with Nile red (red) and DAPI (blue) to detect intracellular lipid droplets (distinct punctate staining as outlined in white boxes) and cell nuclei respectively. For each experiment 4 midguts were included and analysed, figure represents a typical image of the sets. (**b**) Cells were pulse labelled for 30 min with Topfluor (green), a fluorescent cholesterol derivative and treated for 48 h in either 2HPCD or PBS in order to measure cholesterol dynamics. Cell nuclei were stained with DAPI (blue). (**c**) Cellprofiler was used to calculate the number of Topfluor spots per cell in 3 independent replicates for each treatment.

In light of the distinct proteomic profile of the *w*Au-carrying line with respect to proteins involved in lipid transport and homeostasis, cholesterol dynamics and effects of treatment with the cyclodextrin 2HPCD were investigated in *Ae. aegypti* midguts and *Ae. albopictus* cell lines containing *w*Au or *w*Mel, versus *Wolbachia*-negative. As previously reported^18^, *w*Mel-carrying midguts (Fig. 4a, Figure S4) and cells showed an accumulation of cholesterol in lipid droplets (as seen by distinct punctate spots, outlined in white boxes), and 2HPCD treatment in cells led to a dispersal of the accumulated cholesterol (Fig. 4b,c).

**Figure 5:**
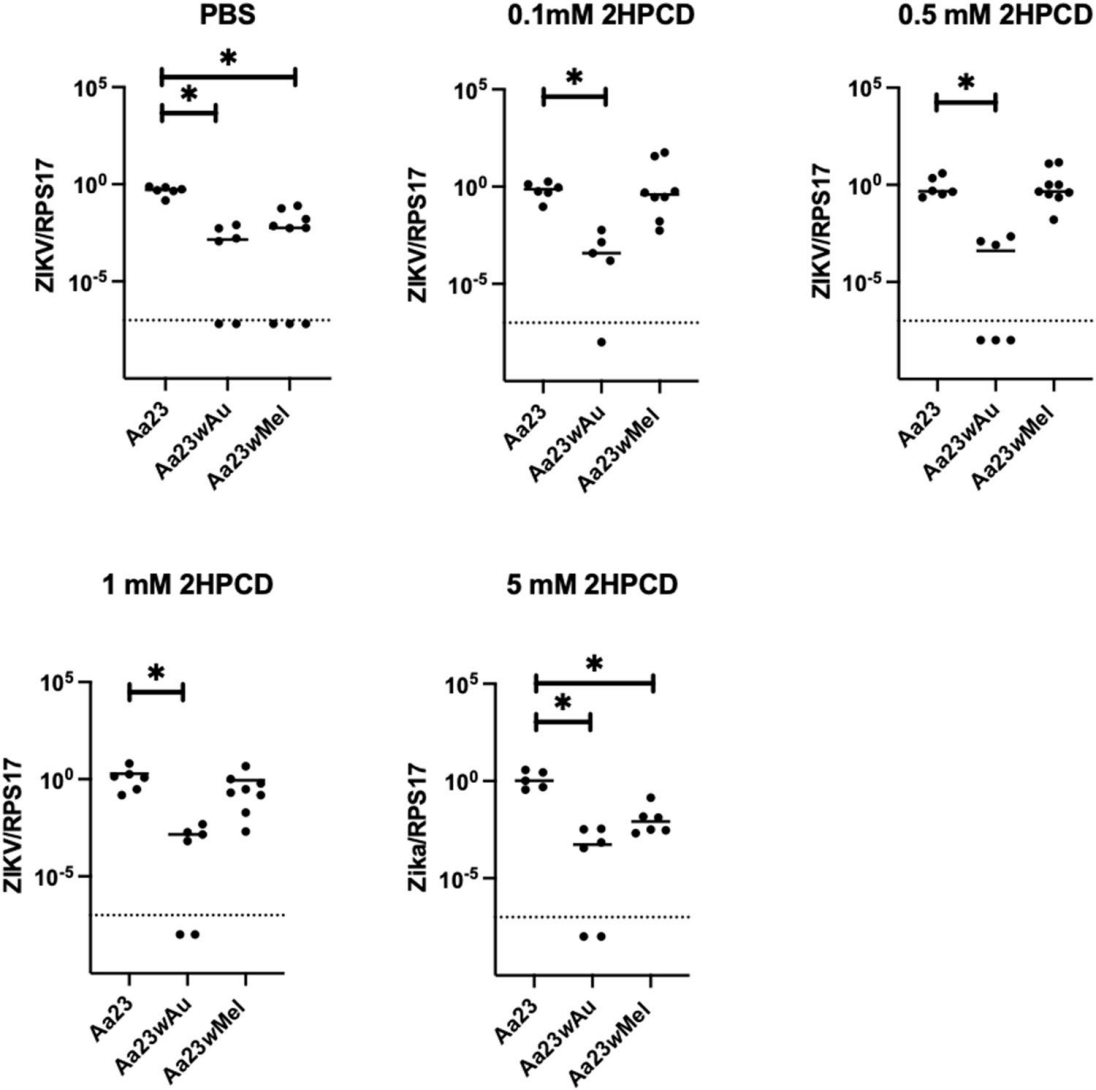
Effect of 2HPCD treatment on ZIKV replication in *Ae. albopictus* cell lines. Cells were treated for 48 h with either PBS or 2HPCD and then infected with ZIKV at an MOI of 1. 72 h post infection total RNA was isolated. qRT-PCR was carried out and data normalised to the mosquito gene RPS17. Tick line represents limit of viral detection on qPCR, therefore anything below represents no virus, each experiment was carried out at least 3 times with a minimum of 2 replicates and combined (*P < 0.05, **P < 0.005 show significant differences in comparisons between the PBS control and each given *Wolbachia* infection status, Mann–Whitney).

However, *w*Au-carrying cells and midguts did not show an accumulation of cholesterol in lipid droplets, while 2HPCD treatment in cells instead led to a significant increase in lipid accumulation. ZIKV replication was completely rescued in *w*Mel-carrying cells treated with 2HPCD, however, there was no replication rescue in *w*Au-carrying cells treated with 2HPCD (Fig. 5). These data clearly recapitulate the differences between *w*Mel and *w*Au lines detected in the proteomic comparisons with respect to perturbations in lipid pathways, and demonstrate that these differences impact virus inhibition.

The inability of 2HPCD to rescue ZIKV replication at 5 mM in *w*Mel cells (Fig. 5) is consistent with previous observations in *Ae. aegypti* cells carrying *w*Mel after infection with DENV^18^. Cyclodextrins can act as cholesterol acceptors and cholesterol donors^43^. If cholesterol:cyclodextrin complexes are pre-formed at the correct molar ratio, they can act as cholesterol donors to cells. In these experiments however, ‘empty’ cyclodextrin is used. It is likely that at lower concentrations, the 2HPCD extracts cholesterol, becomes saturated and is then able to act as a cholesterol donor, in effect re-distributing it to rescue virus replication. At higher concentrations the 2HPCD may instead act as a net cholesterol sink.

## Discussion

The effects of *Wolbachia* in *Ae. aegypti* midgut cells are both profound and diverse between *Wolbachia* strains. The *w*Au and *w*Mel lines showed striking differences in pathways previously demonstrated to have a role in antiviral activity, particularly in the cholesterol and lipid metabolism alterations seen in the *w*Mel line. Changes in multiple pathways are predicted to influence virus replication in *w*Au-carrying *Ae. aegypti*. This does present challenges for functional follow-up experiments, in that the effects of knockdowns or knockouts of particular genes on virus replication are likely to be masked, such that simultaneous knockdowns of multiple genes/pathways will likely be needed to directly demonstrate involvement in the *Wolbachia*-mediated virus inhibition phenotype.

ER trafficking pathways, glycosyltransferases and protein processing in the ER, disrupted specifically by *w*Au, are required for DENV translation and folding of viral proteins. DENV and other arboviruses do not encode glycosyltransferases, which are crucial to several aspects of the viral life cycle. Downregulation of these proteins in insect hosts can have a profound effect on virus binding, replication, protein folding and egress^44^. Protein processing in the ER is likely to be significantly reduced in *w*Au with 12% of the pathway downregulated. DENV replication and assembly relies on these processes in the ER^45^. In order to reach the ER for replication, DENV undergoes clathrin mediated endocytosis^46^; interestingly AAEL014375, a clathrin coat assembly protein, is significantly downregulated in *w*Au but not in *w*Mel or *w*AlbA. Comparison of virus localization after entry and the dynamics of viral replication may help clarify the mechanistic differences further. In *w*AlbA protein export and endocytosis were increased and this may facilitate virus entry.

KEGG pathway analyses revealed that a number of pathways involved in RNA biogenesis, translation and RNA recognition were significantly dysregulated in *w*Au midgut cells. Given the requirement for DENV and other arboviruses to replicate using host machinery, the disruption of these pathways is noteworthy. Flaviviruses in particular are known to hijack the RNA degradation and surveillance pathways in order to replicate^47^. The RNA binding protein Musashi, upregulated in *w*Au, for example is known to bind the 3′UTR of ZIKV and prompt replication/translation, and has been linked to pathogenicity^48^. The ribonuclear protein La (AAEL003664), is upregulated in the *w*Au line, downregulated for *w*AlbA and not significantly dysregulated for *w*Mel. DENV infection causes a re-localisation of the protein and it is found to inhibit replication in a dose dependent manner^36^. The RNA decay pathway has been implicated in antiviral activity of *w*Mel; it has been shown that *w*Mel*-*mediated antiviral activity against DENV in *Ae. aegypti* cells can be reduced by decreasing the levels of XRNI, a key protein involved in RNA decay^49^. There is no increase in XRNI expression associated with *w*Mel, suggesting a functional change rather than a simple increase in protein availability and thus degradation of viral RNA. Therefore, there is a suggestion that RNA decay may play a part in *w*Mel antiviral activity and assessing the effect of deletion of XRNI on *Wolbachia* density would be useful to further investigate this. XRNI depletion in *w*Au lines may also be of interest to determine the effect on antiviral activity, *Wolbachia* titres and the RNA decay pathway.

Host transcriptome analysis in *Drosophila melanogaster* naturally carrying *w*Mel shows that nucleotide metabolism, RNA binding and processing and translation, and transcription are perturbed, similar to *w*Au in this study^50^. This indicates that the host background is also important when determining host interactions and that assessing single tissues and whole organisms may produce different results. Interestingly *w*Mel also appears to have an effect on gene splicing^50^; therefore as *w*Au perturbs the spliceosome it would be interesting to look at transcript profiles in *w*Au and *w*Mel-carrying mosquitoes.

The results presented here indicate that there are clear mechanistic differences underlying antiviral activity between *Wolbachia* strains in terms of the perturbations to lipid and cholesterol transport and intracellular distribution that underpin virus inhibition in *w*Mel, but not in *w*Au. The presence of different mechanisms suggests that, should DENV resistance evolve to counteract the *w*Mel and/or *w*AlbB strains of the symbiont currently being used for DENV control, this resistance may not necessarily function against all *Wolbachia* strains. Experiments using field-caught strains of *Ae. aegypti* carrying introduced *w*Mel or *w*AlbB that have been under field selection for extended periods, found that both strains maintained high levels of transmission inhibition when challenged with DENV isolates^2,51^, but longer-term monitoring is needed. While virus mutations that allow escape from *Wolbachia* antiviral activity have not been reported^52,53^, the ability of arboviruses to rapidly evolve means this is a potential risk to the long-term efficacy of *Wolbachia* DENV control programs. Therefore, the sequential use of *Wolbachia* strains with different antiviral mechanisms could be an important way to counteract viral evolution and maintain the long-term effectiveness of *Wolbachia*-mediated DENV control.

## Methods

### Mosquito rearing and cell work

Mosquito colonies were maintained at standard 27 °C and 70% relative humidity with a 12-h light/dark cycle. Lines have been described previously^8,14^. *Wolbachia*-free lines consist of the original line which *Wolbachia* was transferred into, giving all lines the same genetic background. Tetracycline cured lines were not used as the removal of other bacteria from the lines may have resulted in a skewed proteome not related to the presence or absence of *Wolbachia*. Further to this there is currently no data on the long-term effect of *Wolbachia* on midgut proteomes and if these changes persist after removal of *Wolbachia.* The *w*Mel and *w*AlbA-carrying *Ae. aegypti* lines were simultaneously recovered from a triple-infected (*w*AlbA*w*AlbB*w*Mel) Malaysian *Ae. aegypti* line that displayed incomplete maternal transmission of the superinfection to progeny^54^. The *w*Au line was generated by transferring cytoplasm from *Drosophila simulans* into the Malaysian *Ae. aegypti* laboratory strain. The *w*AlbA, *w*Mel and *w*Au lines were generated within 12-months of each other. Over the initial five generations post transinfection generation, the progeny of initial *Wolbachia*-carrying G0 isofemales (and subsequently of groups of G1–G5 progeny) were sexed and females back-crossed to males from the wild-type Malaysian colony with individualisation for ovisposition and *Wolbachia* screening. Hence, each line had five generations of backcrossing into the wild-type colony, which resulted in colonies of > 200 individuals by G4 and crosses involving transinfected females and wt males numbering in the hundreds of individuals. The *Wolbachia*-carrying lines were therefore expected to have high levels of genetic homogeneity with each other, and the wild-type colony. Age matched (4 days old), mosquitoes were injected in the thorax with 414 nl of 10 mM 2HPCD (this concentration was chosen in line with previously published in vivo experiments in mice and humans^55,56^ using Nanoject II (Drummond Scientific, Pennsylvania, USA) hand-held microinjector, with a pulled glass capillary. 48 h after injection the mosquitoes were blood fed using a Hemotek artificial blood-feeding system (Hemotek, UK) using defibrinated sheep blood (TCS Biosciences, UK). Mosquitoes were allowed to recover for 72 h before midguts were dissected and stained as described below.

In Aa23 (*Aedes albopictus*) cells which had been cleared of *Wolbachia*, *w*Mel and *w*Au strains were introduced from *Drosophila simulans* STCP lines^57^ as follows: Aa23 cells were plated the day before in a 96-well plate. For each *Wolbachia* strain to be transferred, around 200 mated *Drosophila* flies were placed in a BugDorm rearing cage (W17.5 × D17.5 × H17.5 cm) with a Petri dish containing grape agar (3% agar, 1% sucrose, 25% grape juice, water) and a spot of yeast paste in the centre to stimulate egg-laying. After 1 h, around 500 *Drosophila* eggs were collected from the agar plate with a brush and rinsed in sterile water. Eggs were further dechorionated and surface-sterilized in 2.5% bleach for 2 min, 70% ethanol for 5 min twice and were rinsed in sterile water three times. Sterilized eggs were transferred to a 1.5 ml Eppendorf tube, resuspended in PBS and homogenized with a sterile pestle. The egg homogenate was centrifuged at 2500*g* for 10 min at 4 °C to remove cellular debris and the supernatant was filtered through a 5 μm and a 2.7 μm Millex syringe filters. The filtered homogenate was finally centrifuged at 18,500*g* for 5 min at 4 °C to pellet the bacteria. The bacterial pellet was resuspended in 100 µl Schneider's Insect Medium with 10% FBS and overlaid onto the Aa23 cells. Finally, the cell plate was centrifuged at 2500*g* for 1 h at 15 °C. In the following days, fully confluent cells were serially passaged from the 96-well plate, to 48, 24 and 12-well plates. Cells were later maintained in 25 cm^3^ flasks with Schneider’s Insect Medium with 10% FBS at 28 °C. Cells were checked regularly for *Wolbachia* density using quantitative PCR as described previously^8^. Density at time of experiments can be seen in Figure S5.

### Virus infection in cells

A549 cells stably expressing bovine viral diarrhea virus NPro^116^ cells (A549-Npro) were used to propagate ZIKV as described in^58^. For ZIKV infection, Aa23 cells were plated out in 24 well plates at a density of 5 × 10^5^/ml and left to settle for 24 h. After 24 h various concentrations of 2HPCD or PBS was added at varying concentrations and incubated for 48 h in Schneider’s Insect Medium supplemented with 10% FCS. Medium was then removed and ZIKV (PRVABC59-strain) added at a multiplicity of infection of 1 in fresh media. Cells were collected at 72 h post infection. Following the removal of medium Trizol (ThermoFisher UK) was added, and RNA was extracted following manufacturer’s protocol. cDNA was synthesised using the All-In-One cDNA Synthesis SuperMix (Biotools, TX, USA). ZIKV was quantified using ZIKV 835 and ZIKV 911c primers (ZIKV-835: TTGGTCATGATACTGCTGATTGC, ZIKV-911c: CCTTCCACAAAGTCCCTATTGC). Values were normalised to the RpS17 mosquito gene (Rps17-F: CACTCCCAGGTCCGTGGTAT, Rps17-R: GGACACTTCCGGCACGTAGT) as reference by relative expression (Pfaffl method^59^). qPCR was carried out on a Rotor Gene Q machine (Qiagen) using 2 × qQuantiNova SYBR. The following program was used to run the qPCRs: 95 °C for 5 min, 40 × cycles of 95 °C for 15 s and 60 °C for 30 s, followed by a melt-curve analysis.

### Virus infection in mosquitoes

For oral feeding of Semliki Forest virus, sub-type C (catalogue number 1112041v) was obtained from Public Health England culture collections. SFV was propagated on C6/36 cells and fed, as above, at a final concentration of 1.78 × 10^7^ FFU/ml. Females were fed 5 days post adult emergence and 10 days post infectious bloodmeal, heads/thoraxes and abdomens were dissected and placed in Trizol (Thermo Fisher, UK). Reverse-transcriptase qPCR was carried out as detailed above. SFV primers (SFV4-F CGCATCACCTTCTTTTGTG, SFV4-R CCAGACCACCCGAGATTT).

### Staining and imaging

Following dissection, midguts were fixed with Fixative solution [ThermoFisher UK (United Kingdom)] for 10 min, followed by 3 washes in PBS. Midguts were then incubated in Nile red (Sigma) stain at 0.1 μg/ml for 40 min, followed by three PBS washes and mounted in ProLong™ Gold Antifade Mountant with DAPi (ThermoFisher UK). Aa23 cells were pulse labelled with the cholesterol derivative Topfluor as previously described^18^ and treated as above with either 2HPCD (2 hydroxypropyl β cyclodextrin) or PBS. All images were then acquired using a Zeiss LSM 880 confocal microscope (Zeiss) with a 63× objective for cells and 20× objective for midguts. Nile red was imaged using a 514 nm laser, excitation was measured at 646 nm, to allow recording of polar and non-polar lipids. Nuclei stained with DAPI were imaged using a 405 nm laser detector. TopFluor was imaged using a 488 nm laser, with GaAsP detectors. All settings were obtained by first imaging uninfected Aa23 *Wolbachia*-negative cells incubated in PBS as a standard control. For midguts settings were obtained by first imaging wt, PBS as a standard control. Quantification was carried out by imaging 3 independent × 64 images from 3 independent wells on a 24-well optical plate. Images were analysed using Cell Profiler. A global image threshold was set using the Otsu method and images were analysed in order to identify the number of nuclei and the number of green spots corresponding to TopFluor staining. Data are presented as the number of spots per cell. Large crystals in cells due to precipitated Topfluor were masked from images to ensure only intracellular fluorescence was measured.

### Proteomics sample preparation

Proteomic analysis was carried out on midguts from age matched (10 days old), non-bloodfed, female mosquitoes [*w*Au, *w*AlbA and Wild-type (no *Wolbachia*)]. All *w*Mel data was previously sampled^18^ using the same equipment, methodologies and investigators. Each biological replicate consisted of pooled midguts from 20 individuals, 5 biological replicates were analysed for each *Wolbachia* infection type. Each biological replicate was lysed in 200 µl 8 M urea 50 mM triethylammonium bicarbonate (TEAB) supplemented with 1 × protease inhibitor (Roche). Midguts were sonicated for 3 cycles of 15 s yielding approximately 100 µg of protein from each pool as measured by BCA assay (Thermo Scientific). Samples were reduced with 5 mM DTT for 30 min at 50 °C then alkylated with 15 mM IAA for 30 min at RT. Urea was diluted to a final concentration of 1.5 M and trypsin/Lys-C (Promega) added to a ratio of 25:1 protein:trypsin. After overnight digestion at 37 °C, the digest was acidified with 0.5% trifluoroacetic acid (v:v) and centrifuged at 18,000*g* for 7 min. Digested peptides were desalted using 50 mg C18 cartridges (Phenomenex Strata) and dried down. Peptides were resuspended in 50 mM TEAB and labelled with a TMT 10plex kit (Thermo Scientific). From each biological replicate, 6 µg of peptide was taken, this was pooled and labelled with the 131 TMT channel to create a common pool reference channel enabling relative quantification across all 15 samples. 44 µg of peptide from each biological replicate was labelled, replicates were pooled into two groups. Each group was fractionated by high pH reversed phase fractionation according to manufacturers instructions (Thermo Scientific).

### Mass spectrometry

Peptides from midgut samples were resuspended in 0.1% formic acid and loaded onto an UltiMate 3000 RSLCnano HPLC system (Thermo) equipped with a PepMap 100 Å C18, 5 µm trap column (300 µm × 5 mm, Thermo) and a PepMap, 2 µm, 100 Å, C18 Easy Nanocapillary column (75 μm × 150 mm, Thermo). The trap wash solvent was 0.05% (v:v) aqueous TFA and the trapping flow rate was 15 µl/min. The trap was washed for 3 min before switching flow to the capillary column. Separation used gradient elution of two solvents: solvent A, aqueous 1% (v:v) formic acid; solvent B, aqueous 80% (v:v) acetonitrile containing 1% (v:v) formic acid. The flow rate for the capillary column was 300 nl/min and the column temperature was 40 °C. The linear multi-step gradient profile was: 3–10% B over 8 min, 10–35% B over 125 min, 35–65% B over 50 min, 65–99% B over 7 min and then proceeded to wash with 99% solvent B for 4 min. The column was returned to initial conditions and re-equilibrated for 15 min before subsequent injections.

The nanoLC system was interfaced with an Orbitrap Fusion hybrid mass spectrometer (Thermo) with an EasyNano ionisation source (Thermo). Positive electrospray ionisation (ESI)-MS, MS2 and MS3 spectra were acquired using Xcalibur software (version 4.0, Thermo). Instrument source settings were: ion spray voltage, 1900 V; sweep gas, 0 Arb; ion transfer tube temperature, 275 °C. MS1 spectra were acquired in the Orbitrap with: 120,000 resolution, scan range: m/z 380–1500; automatic gain control (AGC) target, 2e5; max fill time, 50 ms. Data-dependant acquisition was performed in top speed mode using a 4 s cycle, selecting the most intense precursors with charge states > 1. Dynamic exclusion was performed for 50 s post-precursor selection and a minimum threshold for fragmentation was set at 3e4. MS2 spectra were acquired in the linear ion trap with: scan rate, turbo; quadrupole isolation, 1.2 m/z; activation type, collision-induced dissociation; activation energy: 35%; AGC target, 1e4; first mass, 120 m/z; max fill time, 50 ms. MS3 spectra were acquired in multi notch synchronous precursor mode (SPS3), selecting the 5 most intense MS2 fragment ions between 400 and 1000 m/z. SPS3 spectra were measured in the Orbitrap mass analyser using: 50,000 resolution, quadrupole isolation, 2 m/z; activation type, HCD; collision energy, 65%; scan range: m/z 110–500; AGC target, 5e4; max fill time, 86 ms. Acquisitions were arranged by Xcalibur to inject ions for all available parallelisable time.

### MS data analysis

TMT data peak lists were converted from centroided .raw to .mgf format using Mascot Distiller (version 2.6.1, Matrix Science) and MS3 spectra were concatenated into their parent MS2 spectra for database searching. Mascot Daemon (version 2.5.1, Matrix Science) was used to combine .mgf files and search against a subset of the UniProt database containing *Ae. aegypti* and *Wolbachia w*Mel proteins (17,811 sequences) using a locally running copy of the Mascot program (Matrix Science Ltd, version 2.5.1). Search criteria specified: enzyme, trypsin; fixed modifications, carbamidomethyl (C), TMT10plex (N-term, K); variable modifications, oxidation (M); peptide tolerance, 5 p.p.m.; MS/MS tolerance, 0.5 Da; Instrument, ESI-TRAP. The Mascot .dat result file was imported into Scaffold Q+ (version 4.7.5, Proteome Software) and a second search run against the same database using X!Tandem was run. Protein identifications were filtered to require a maximum protein and peptide FDR of 1% with a minimum of two unique peptide identifications per protein. Protein probabilities were assigned by the Protein Prophet algorithm. Proteins that contained similar peptides and could not be differentiated based on MS/MS analysis alone were grouped to satisfy the principles of parsimony. Proteins sharing significant peptide evidence were grouped into clusters. Quantification of relative protein abundance was calculated from TMT reporter ion intensities with Scaffold Q+ using the common pool reference channel. TMT isotope correction factors were taken from the document supplied with the reagents by the manufacturer.

Normalised log2 transformed protein intensities were analysed with Limma^60^ to determine significant differences between sample groups at a 5% False Discovery Rate, options ‘trend’ and ‘robust’ were enabled in the empirical Bayes procedure. Multiple testing correction was carried out according to Benjamini & Hochberg. For the KEGG pathway bubble plot, significantly dysregulated proteins detected by Limma were submitted to StringDB to detect over-represented pathways. Significantly regulated proteins from *w*AlbA (p < 0.0135), *w*Au (p < 0.01559), *w*Mel (p < 0.01463) midguts were split into downregulated and upregulated groups for each *Wolbachia* type, each group was then submitted to StringDB to calculate enriched KEGG pathways^26^. Further to this, for comparison of *w*Au and *w*AlbA, data was split into 3 criteria as outlined in Fig. S3 for KEGG pathway analysis.

## Supplementary Information

Supplementary Information 1.

Supplementary Information 2.

## Data Availability

Proteomic raw data is included in supplementary dataset and available at MassIVE dataset MSV000092124. RIC data generated will be available on request from the corresponding authors as this data set is part of a larger dataset awaiting publication. All other data is available on The University of Glasgow Enlighten repository http://dx.doi.org/10.5525/gla.researchdata.1452.
