# Supplementary material for: Differences in proteome perturbations caused by the Wolbachia strain wAu suggest multiple mechanisms of Wolbachia-mediated antiviral activity
Source: Sci Rep. 2023 Jul 20;13:11737. doi: 10.1038/s41598-023-38127-4 (PMC10359319; doi:10.1038/s41598-023-38127-4)
Supplement: Supplementary file 1 — Supplementary Information 1. [file 41598_2023_38127_MOESM1_ESM.pdf]

Supplementary data

Table S1 Comparison of *w*Au/*w*AlbA/wt proteomic analysis and *w*Mel/wt analysis
Comparison of dysregulated proteins involved in Lipid metabolism and ER/protein unfolding response between data sets. Numbers indicate the fold change when compared to the corresponding wt midgut data. Figures greyed out represent those not found to be significant in a given dataset.

**Lipid metabolism**	*w*Mel[1]	*w*Au	*w*AlbA
Niemann-Pick type C2 (AAEL015136)	2.30	0.36	0.60
Niemann-Pick type C2 (AAEL009760)	2.06	0.99	1.27
Sterol carrier protein 2-like 2	1.85	0.81	1.01
Niemann-Pick type C1	1.35	0.76	1.08
Palmitoyltransferase	1.33	0.90	1.05
Lipid droplet associated hydrolase	1.33	0.95	1.19
Sterol carrier protein 2-like 3 variant 1	1.27	0.77	0.66
Putative oxysterol binding protein	1.24	1.02	1.02
Phosphoacetlyglucosamine mutase	1.21	0.92	1.02
Putative cery long-chain acyl-coa synthetase/fatty acid transporter	1.17	0.95	1.10
Glycerol-3-phosphate acyltransferase 3-like	1.15	0.86	1.04
Putative 85kDa calcium-independent phospholipase a2	1.13	0.88	0.95
Putative lipid particle	0.89	1.13	1.11
Sterol carrier protein-X	0.88	1.38	1.20
Acyl-coenzyme A oxidase	0.88	0.99	0.99
Putative fatty acid desaturase	0.82	0.93	0.92
**ER/unfolded protein response**	*w*Mel[1]	*w*Au	*w*AlbA
Vacuolar ATPase assembly integral membrane protein	1.36	n/a	n/a
Alpha-glucosidase	1.26	0.98	0.97
Dolichyl-diphosphooligosaccharide--protein glycosyltransferase subunit stt3b	1.21	0.82	1.08
Olichyl-diphosphooligosaccharide--protein glycosyltransferase dub unit DAD1	1.21	1.08	1.15
tRNA-splicing ligase RtcB	1.20	0.86	1.04
Putative mannose lectin ergic-53	1.16	0.99	1.05
Putative yip1	1.16	0.91	0.97
Reticulon-like protein	1.16	0.79	1.02
Putative proteasome non-atpase 26s subunit	1.13	1.03	0.79
Putative sec23-binding domain of sec16	1.12	0.99	1.04
Heat shock protein	1.11	1.13	1.11
Peptidylprolyl isomerase	1.09	0.90	1.01
Putative transport and Golgi organisation protein 1	1.09	0.90	1.01
Gp210	0.91	1.09	1.01

Table S2 RBPs significantly dysregulated in *w*Au midguts compared to *Wolbachia*-free midguts.

Proteins significantly dysregulated in wAu midguts, identified as RBPs through comparison to RIC dataset are shown. Those already known to bind DENV RNA are highlighted. Permutation Test Benjamini-Hochberg

AlternateA1:I45 ID	(p <0.01559)(wAu / WT)	Log2Fold Change (wAu / WT)	(p < 0.01352)(wAlbA / WT)	Log2 Fold Change (wAlbA / WT)	(p < 0.01334)(wt / wMel	Log2 Fold Change(wmel / wt)	Known to bind DENV
AAEL004978+A2:A94	0.0001	-0.55	0.00025	-0.5	0.001	-0.25	
AAEL003835	0.0001	-0.21	0.57	-0.02	0.0001	0.27	
AAEL003916	0.0001	-0.2	0.23	-0.04	0.002	0.21	
AAEL013139	0.0001	-0.18	0.00077	-0.11	x	x	
AAEL000944	0.008	-0.08	0.66	0	x	x	
AAEL012585	0.00041	0.05	0.001	-0.05	0.0001	-0.2	
AAEL001421	0.004	0.05	0.98	0	x	x	Binds DENV and ZIKV in humans
AAEL005722	0.001	0.06	0.0087	-0.05	0.0001	-0.13	Binds DENV NS5
AAEL006577	0.009	0.07	0.0001	0.14	x	x	
AAEL006794	0.00059	0.07	0.0001	0.11	x	x	
AAEL012686	0.003	0.07	0.081	0	x	x	
AAEL013249	0.003	0.08	0.0001	0.12	0.0001	0.14	
AAEL009326	0.013	0.08	0.0001	0.15	x	x	
AAEL013359	0.004	0.08	0.004	-0.05	0.009	-0.07	
AAEL010018	0.001	0.08	0.1	0.03	x	x	
AAEL002488	0.001	0.09	0.0001	0.11	0.0001	0.22	
AAEL001352	0.0001	0.09	0.002	-0.04	x	x	
AAEL009859	0.00083	0.09	0.027	0.05	0.008	0.07	
AAEL009151	0.003	0.09	0.22	0.01	x	x	
AAEL011447	0.00051	0.09	0.55	0.03	x	x	
AAEL009496	0.00022	0.09	0.025	-0.05	0.0001	-0.24	
AAEL008248	0.001	0.1	0.003	0.11	x	x	
AAEL011698	0.002	0.1	0.0001	0.14	x	x	
AAEL008073	0.00087	0.1	0.12	0.11	0.008	0.08	
AAEL000750	0.00044	0.1	0.093	0.05	x	x	
AAEL001166	0.001	0.1	0.24	-0.04	x	x	
AAEL004699	0.0001	0.11	0.001	-0.02	0.0001	-0.13	binds DENV RNA
AAEL008220	0.00056	0.11	0.00019	-0.1	0.011	-0.13	
AAEL011282	0.005	0.11	0.0001	-0.1	0.0001	-0.24	prM DENV binding
AAEL005947	0.00038	0.11	0.004	-0.04	x	x	
AAEL005744	0.0001	0.11	0.7	-0.02	x	x	
AAEL010248	0.00074	0.11	0.12	-0.02	x	x	
AAEL013968	0.0001	0.12	0.011	0.05	x	x	binds DENV RNA
AAEL003514	0.001	0.12	0.01	-0.08	x	x	
AAEL013982	0.0001	0.12	0.56	0.02	x	x	
AAEL005629	0.002	0.12	0.17	-0.01	0.002	-0.16	
AAEL001037	0.001	0.12	0.21	-0.05	x	x	
AAEL003385	0.003	0.13	0.007	0.16	x	x	
AAEL004415	0.001	0.13	0.93	-0.02	x	x	
AAEL009309	0.0017	0.13	0.063	-0.08	x	x	
AAEL009287	0.0065	0.14	0.77	0	0.001	-0.15	
AAEL004859	0.0001	0.14	0.67	0.01	x	x	
AAEL013144	0.0001	0.14	0.58	0	x	x	
AAEL000385	0.001	0.14	0.057	-0.1	x	x	
AAEL003664	0.0001	0.16	0.003	-0.05	x	x	binds DENV 3'UTR
AAEL001356	0.0001	0.16	0.87	0	0.002	-0.11	
AAEL001216	0.0001	0.16	0.56	0.01	x	x	
AAEL004017	0.0001	0.16	0.55	0.01	x	x	
AAEL008100	0.002	0.16	0.41	-0.03	x	x	
AAEL010467	0.00018	0.16	0.12	-0.04	x	x	
AAEL011150	0.004	0.16	0.048	-0.12	x	x	
AAEL005544	0.001	0.17	0.003	0.1	x	x	
AAEL009067	0.0001	0.17	0.00037	0.1	x	x	
AAEL010042	0.0001	0.17	0.0001	0.19	x	x	
AAEL006887	0.0001	0.17	0.024	0.08	x	x	
AAEL014959	0.0001	0.17	0.26	0.04	x	x	binds DENV RNA
AAEL012964	0.0001	0.17	0.62	-0.01	x	x	
AAEL012980	0.00081	0.17	0.93	-0.01	x	x	
AAEL012312	0.0001	0.18	0.0001	0.14	0.0001	-0.12	
AAEL007042	0.0001	0.18	0.83	0.01	x	x	
AAEL009224	0.003	0.18	0.64	0.04	x	x	
AAEL001350	0.0001	0.19	0.001	0.11	x	x	
AAEL003801	0.0001	0.19	0.001	0.15	x	x	
AAEL008169	0.0001	0.19	0.001	0.15	x	x	
AAEL011925	0.0001	0.19	0.0001	0.2	x	x	
AAEL010340	0.0001	0.19	0.32	0.01	x	x	
AAEL012025	0.0001	0.2	0.0001	0.19	0.0016	0.15	
AAEL013116	0.0001	0.2	0.0001	0.23	x	x	
AAEL013771	0.00057	0.2	0.0001	0.26	x	x	
AAEL009217	0.00042	0.2	0.18	0.1	x	x	
AAEL007762	0.0001	0.21	0.0001	0.15	x	x	
AAEL011340	0.001	0.21	0.36	0.05	x	x	
AAEL006876	0.00064	0.22	0.002	-0.11	0.006	-0.28	
AAEL003944	0.0001	0.22	0.62	0.02	0.0001	-0.26	
AAEL005356	0.0001	0.22	0.014	0.11	x	x	
AAEL010007	0.008	0.22	0.43	0.08	x	x	
AAEL004643	0.0001	0.23	0.0001	0.22	0.0001	0.23	
AAEL007565	0.0001	0.23	0.0001	0.13	0.014	0.05	
AAEL008257	0.0001	0.23	0.003	0.06	x	x	binds DENV RNA
AAEL008601	0.0001	0.23	0.0001	0.23	x	x	
AAEL002832	0.001	0.23	0.003	-0.21	0.004	-0.14	
AAEL012621	0.0001	0.23	0.076	0.05	0.001	-0.15	
AAEL012826	0.0001	0.25	0.12	0.07	x	x	
AAEL004455	0.0001	0.25	0.51	-0.04	x	x	
AAEL002418	0.0001	0.26	0.00099	0.27	x	x	
AAEL003142	0.0001	0.26	0.0001	0.18	x	x	binds DENV RNA
AAEL005190	0.0001	0.26	0.00037	0.15	x	x	binds DENV RNA
AAEL002083	0.0001	0.26	0.027	0.09	x	x	
AAEL001506	0.001	0.27	0.0052	0.2	x	x	
AAEL011198	0.0001	0.28	0.0001	0.16	x	x	
AAEL003293	0.0001	0.3	0.013	0.15	x	x	
AAEL013075	0.0001	0.34	0.4	0.05	0.0001	-0.24	
AAEL005515	0.009	0.44	0.73	-0.03	0.008	-1.83	

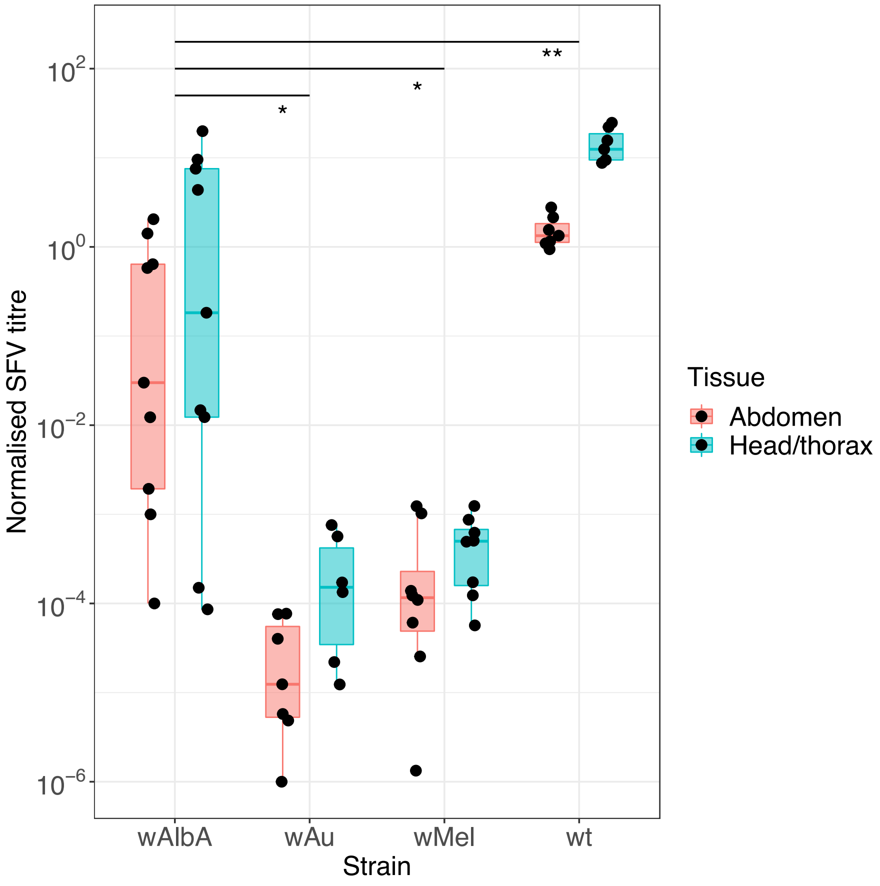

Figure S1 *Wolbachia* inhibition of Semliki Forest virus after infectious blood meal
Semliki Forest virus (SFV) genome copies per host cell following an infectious blood meal 5 days post adult emergence to *Wolbachia*-containing lines and wild-type *Ae. aegypti*. Females were left for 10 days prior to dissection of the abdomen and head/thorax and total RNA was extracted. Virus quantification was carried out by reverse-transcriptase qPCR with virus levels normalised to host RNA levels using RpS17 as a house-keeping gene. PCR was carried out on cDNA from 9, 7, 8 and 8 females from the *w*AlbA, *w*Au, *w*Mel and wt lines respectively. Statistical analysis was performed using a one-way ANOVA with a Dunnett's post-hoc test.

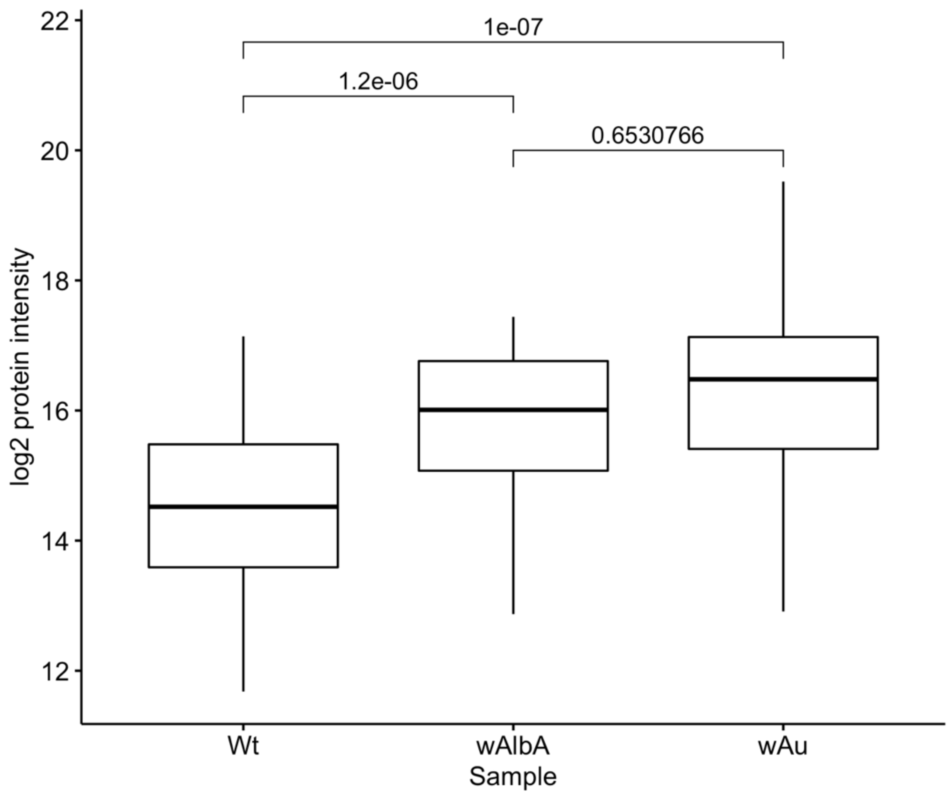

Figure S2 Median intensity of *Wolbachia* proteins in *Aedes aegypti* midguts. Intensities of TMT reporter ions derived from *Wolbachia* proteins was compared between mosquito lines. *Wolbachia* protein intensity was statistically compared using ANOVA followed by Tukey-HSD test, adjusted p-values are shown for each pairwise comparison. Signal seen in the Wt channel is solely background intensity due to noise/co-isolation of non-*Wolbachia* derived peptides during fragmentation and trace isotopes in the TMT channel.

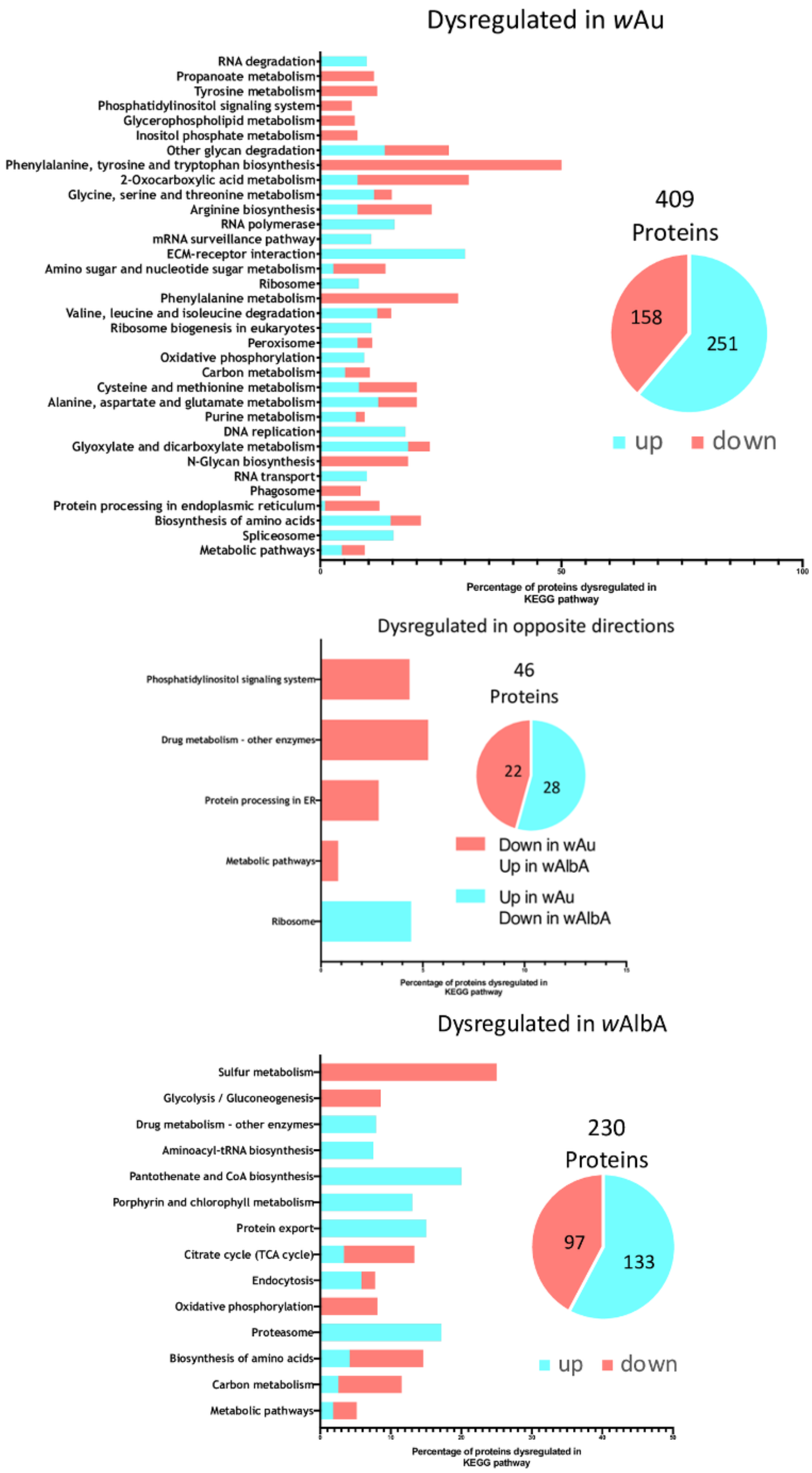

Figure S3 Separation into criteria Proteins significantly dysregulated between *w*Au and *w*AlbA when compared to wt midguts were split into 3 criteria and KEGG pathway analysis carried out in order to determine pathways significantly enriched in each criterion (FDR adjusted p-value <0.05).

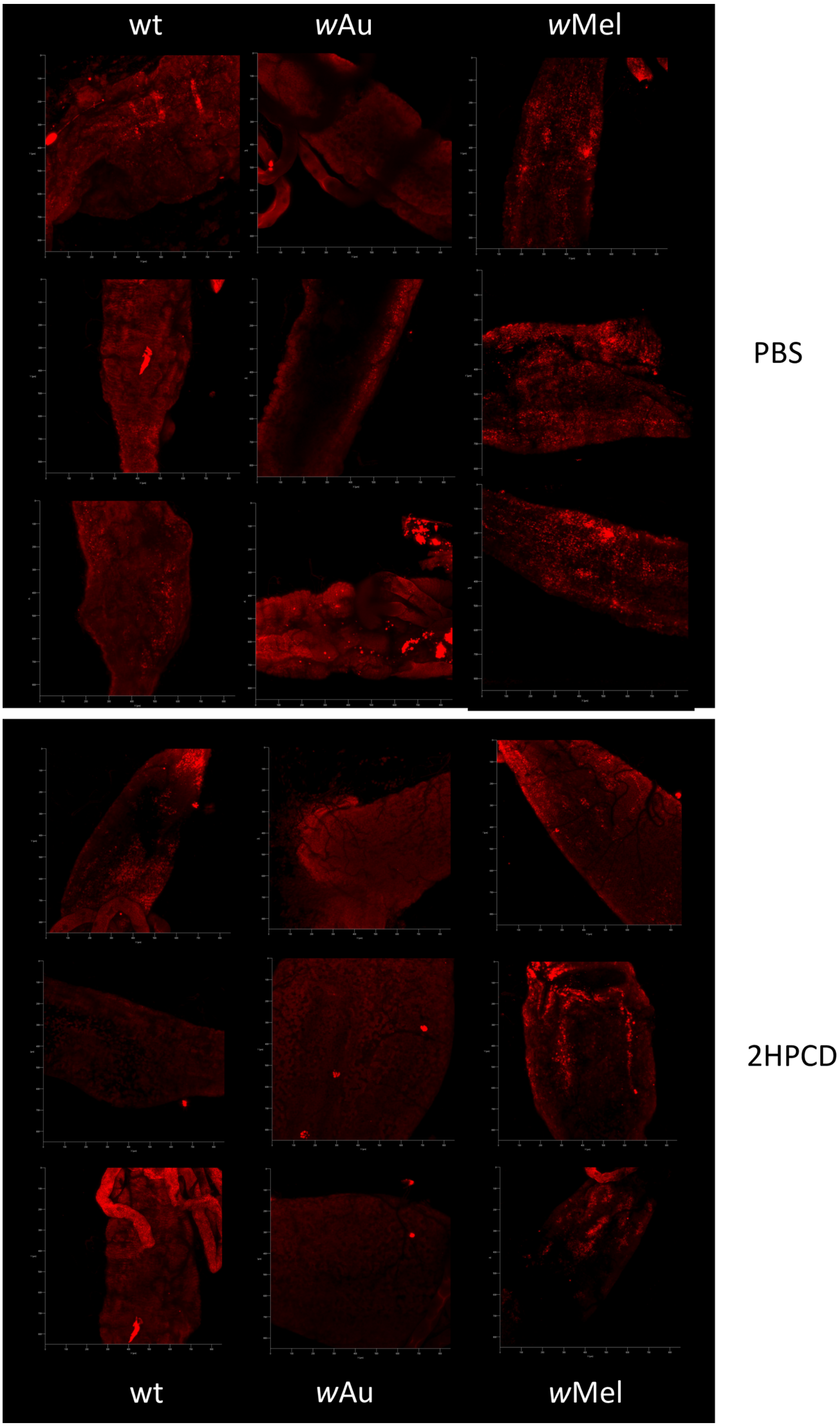

Figure S4 Nile red lipid staining of bloodfed mosquito midguts
Aedes aegypti mosquitoes were injected with 10 mM of 2HPCD or PBS, left to recover for 2 days before a bloodmeal was given. 72hr post bloodmeal midguts were dissected, fixed and stained with Nile red to detect intracellular lipid droplets (distinct punctate staining) For each experiment 3

midguts were included and analysed. Figure shows maximum intensity projection of a whole midgut generated via a Z-stack scan.

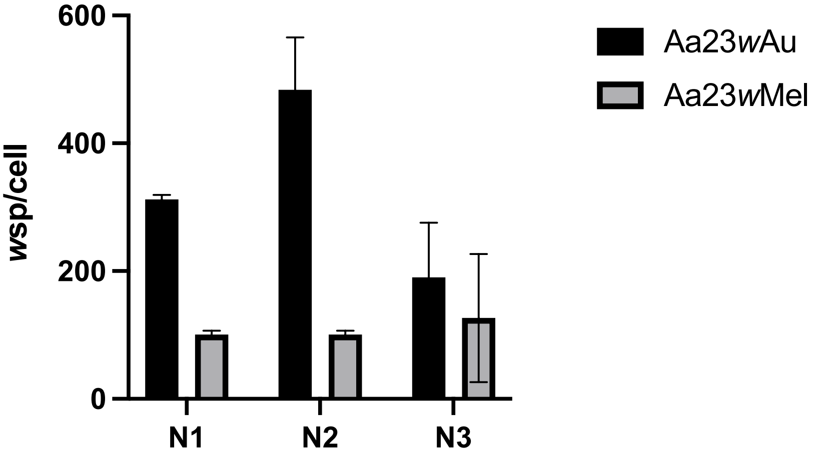

Figure S5 Density of *Wolbachia* in cell lines prior to Zika virus challenge Density of *Wolbachia* within cell lines was measured prior to infection with Zika virus. *Wolbachia* quantification was carried out by qPCR. Levels of target sequence were normalized against the HTH house-keeping gene. X-axis label corresponds to each of the 3 independent replicates carried out in Zika challenge experiments. Delta-delta Ct values were used in order to calculate the number of Wolbachia genome copies per cell.
